# Computed tomography Osteoabsorptiometry: Review of bone density, mechanical strength of material and clinical application

**DOI:** 10.3389/fbioe.2023.1066709

**Published:** 2023-03-27

**Authors:** Guanghua Xu, Qiuyuan Wang, Zhiqiang Li, Tingrui Wu

**Affiliations:** ^1^ Orthopedic Center, Affiliated Hospital of Guangdong Medical University, Zhanjiang, China; ^2^ Guangdong Engineering Research Center for Translation of Medical 3D Printing Application, Guangdong Provincial Key Laboratory of Medical Biomechanics, National Key Discipline of Human Anatomy, School of Basic Medical Sciences, Southern Medical University, Guangzhou, China; ^3^ Institute of Sport and Exercise Medicine, North University of China, Taiyuan, China; ^4^ Graduate School of Beijing University of Chinese Medicine, Beijing, China; ^5^ School of Physical Education, North University of China, Taiyuan, China

**Keywords:** computed tomography, computed tomography osteoabsorptiometry, post-processing, biomechanical, stress, high density area

## Abstract

Computed Tomography (CT) imaging is an effective non-invasive examination. It is widely used in the diagnosis of fractures, arthritis, tumor, and some anatomical characteristics of patients. The density value (Hounsfield unit, HU) of a material in computed tomography can be the same for materials with varying elemental compositions. This value depends on the mass density of the material and the degree of X-ray attenuation. Computed Tomography Osteoabsorptiometry (CTOAM) imaging technology is developed on the basis of CT imaging technology. By applying pseudo-color image processing to the articular surface, it is used to analyze the distribution of bone mineralization under the articular cartilage, evaluate the position of prosthesis implantation, track the progression of osteoarthritis, and determine the joint injury prognosis. Furthermore, this technique was combined with indentation testing to discuss the relationship between the high bone density area of the articular surface, the mechanical strength of the bone, and the anchorage stability of the implant, in addition to the study of the relationship between mechanical strength and bone density. This narrative study discusses the pre- and postoperative evaluation of medical device implantation position, orthopedic surgery, and the clinical treatment of bone injury and degeneration. It also discusses the research status of CTOAM technology in image post-processing engineering and the relationship between bone material and mechanical strength.

## 1 Introduction

Bone is an organ with active metabolism, which is constantly reshaped throughout life. Bone remodeling involves the removal of mineralized bone by osteoclasts, the formation of bone matrix by osteoblasts, and then the formation of mineralization. The reconstruction cycle includes three consecutive stages: absorption, conversion and post formation (HADJIDAKIS and ANDROULAKIS, 2006). Bone remodeling is to adjust the bone structure to meet the changing mechanical needs, repair minor damage in the bone matrix, and prevent the accumulation of old bone. Therefore, the bone turnover sequence needs to be strictly controlled by the body. If the body has serious abnormal bone absorption or bone remodeling imbalance, there would be rapid bone loss and bone growth in the body, especially for the rehabilitation of patients after joint orthopedic surgery, the probability of osteoporosis and fracture.

Computed tomography (CT) is a cross-sectional 2D image and 3D reconstruction model method based on the photoelectric signal conversion formed by the absorption and attenuation effect between X-ray photons and human tissue, and formed by a post-processing system. The attenuation value (HU) corresponding to bone tissue is converted to the equivalent value of bone tissue by calibrating the mold body with hydroxyapatite ceramic and special analysis software. CT has been widely used for diagnosis in clinical practice. It is believed that fracture risk is affected by changes in the structure of specific bone sites, including the different functional roles of cortical bone and trabecular bone, which proves that the efforts to analyze these entities alone are reasonable (Kemp et al., 2014).

**FIGURE 1:**
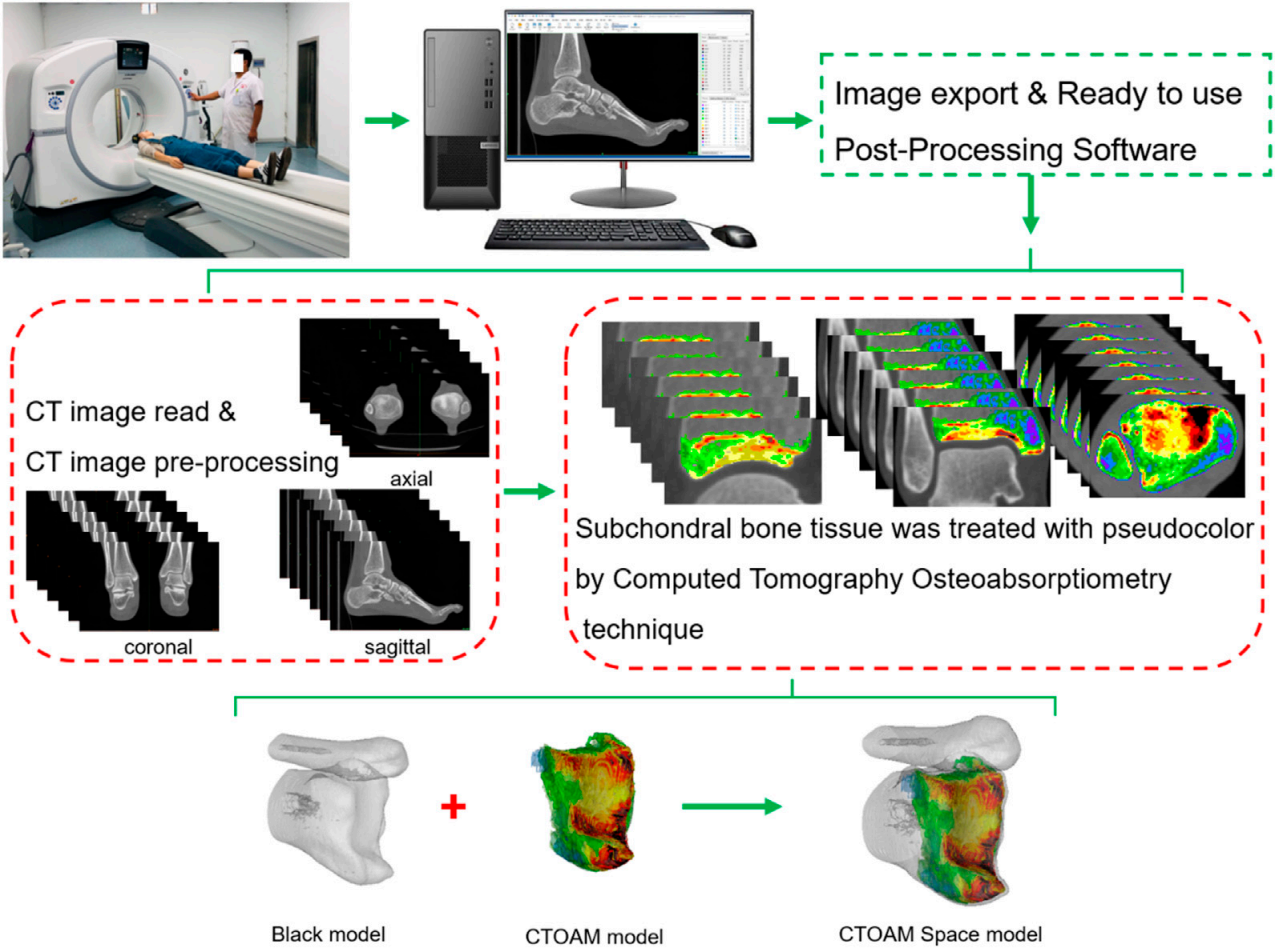
The computed tomography Osteoabsorptiometry (CTOAM) technical route model diagram.

The computed tomography Osteoabsorptiometry (CTOAM) technology is a CT image processing technology that uses the absorption attenuation effect between X-ray photons and human bone tissue to form a topographic map of bone tissue specificity with different densities. It was invented by Muller Gerbl et al. in the early 1989s (Müller-Gerbl et al., 1989). According to Wolff’s law, bones adapt to functional needs through remodeling, which can also reflect the distribution of resultant forces acting on the bone. This method does not focus on the quantitative calculation of absolute value. The key of this method is to present the difference in relative density on the surface and inside the joint. Due to its relatively greater stiffness and strength than the covered articular cartilage, the subchondral bone absorbs most of the mechanical force transmitted by the double joint joints and provides mechanical support for the covered articular cartilage (Layton et al., 1988; Madry, 2010). Compared with the relatively slow articular cartilage turnover rate, subchondral bone undergoes faster modeling and remodeling in response to changes in the mechanical environment (Goldring and Bianchi, 2012). By verifying the CT bone mineral density information obtained from anatomical specimens and comparing it with the X-ray bone mineral density information, it was found that the results of bone mineral density between the information of CT and X-ray image were highly similar, and a joint density distribution map was generated in the joint image (in Figure 1.) (Muller-Gerbl et al., 1990a; Muller-Gerbl et al., 1990b). Different from the bone mineral density measurement method in DXA for osteoporosis diagnosis, when using CT for bone mineral density measurement, repeatable results can only be obtained on the subchondral bone plate or dense bone due to various radiological effects (partial volume effect and beam hardening effect). The long-term mechanical stress transmitted in the joint can stimulate the adaptive changes in bone matrix density and mineral salt content in the growth process, which can show the load history, distribution patterns of the subchondral bone density, the place of load areas, and joint sports patterns of articular subchondral bone. Studies have found that mechanical stress acting on the joints can lead to remodeling changes in the subchondral bone of the joint (Oettmeier et al., 1992). The CTOAM was originally used to evaluate the prognosis of osteoarthritis before and after orthopedic surgery. It has also been used for studying the characteristics of the bone and joint in special working groups influenced by extra mechanical stress, such as athletes, physical workers, etc. The stress distribution and stress loading history *in vivo* joints can also be presented through this non-invasive diagnostic technique (von Eisenhart-Rothe et al., 1997; Eckstein et al., 1995a; Eckstein et al., 1995b).

This narrative study discusses the pre- and postoperative evaluation of medical device implantation position, orthopedic surgery, and the clinical treatment of bone injury and degeneration. It also discusses the research status of CTOAM technology in image post-processing engineering and the relationship between bone material and mechanical strength.

## 2 Technical background

The computed tomography Osteoabsorptiometry (CTOAM) technology is a CT image processing technology that uses the absorption attenuation effect between X-ray photons and human bone tissue to form a topographic map of bone tissue specificity with different densities. According to Wolff’s law, bones adapt to functional needs through remodeling, which can also reflect the distribution of resultant forces acting on the bone. This method does not focus on the quantitative calculation of absolute value. The core of this method is to present the difference in relative density on the surface and inside the joint. The long-term mechanical stress transmitted in the joint can stimulate the adaptive changes in bone matrix density and mineral salt content in the growth process, which can show the load history of articular subchondral bone under long-term stress. Studies have found that mechanical stress acting on the joints can lead to remodeling changes in the subchondral bone of the joint (Oettmeier et al., 1992). The non-uniformity of remodeling sites in the joint mainly depends on the initial geometry of the joint surface, the degree of stress load, the main stress sites and the length of loading history.

## 3 Post-processing applications

The maximum density projection is drawn by extracting the highest signal intensity of each point on the subchondral plate on the projection line. This is a process of converting and compressing a series of voxels on the projection line of a three-dimensional object into a representative pixel in a two-dimensional density map. The reconstructed image is displayed as a gray-scale image, but in fact, there is information about the signal intensity in the saved image. Through Hounsfield unit (HU), that is, the attenuation coefficient used in CT. For better observation, the topographic maps of various densities in CT images are pseudo colored. Finally, the pseudo color topographic map is used for data statistical analysis. As HU is the attenuation coefficient calibrated according to water rather than absolute value, the data difference generated by HU may show different result data according to the difference in CT scanner KV parameters, CT scanner brand, algorithm or inspection conditions, resulting in the lack of repeatability of image measurement data. Of course, under same condition, this error may be reduced to a certain extent as all subjects use the same CT scanner and the same scanning parameters for shooting, measurement and evaluation (Eckstein et al., 1995a; Eckstein et al., 1995b; Rhee and Baek, 2012). Therefore, CTOAM is a kind of image processing and measurement technology with repeatability and high sensitivity.

## 4 Correlation between bone density and mechanical strength

Currently, DXA is used as the primary means to assess bone density and establish a diagnosis of osteoporosis, but it is limited to a two-dimensional assessment. In qCT, however, there is a similar situation in which the absolute value of the density of a region of interest in a specific plane is measured. By measuring BMD from a single plane, DXA and qCT only enable clinicians to obtain a broad understanding of bone mass but cannot provide density distribution data for specific regions of interest. On the other hand, although most 3D techniques measure absolute BMD values in specific regions, including cortical and cancellous bone, the density distribution difference of the region of interest, particularly the density distribution of subchondral bone, can be captured by CT-OAM as a 3D imaging approach.

qCT is a methodology for assessing bone density utilizing phantom quantitative technique. Although CT and qCT use the same X-ray absorption-attenuation technique to evaluate bone density, qCT and CT can expose patients to higher levels of ionizing radiation than DXA. Second, in the clinical diagnosis process, the preoperative assessment of patients often adopts X-ray examination to CT testing. If a specific clinical need exists, clinicians will investigate DXA or qCT. Furthermore, qCT is commonly employed in scientific research, whereas DXA is commonly used for clinical bone density evaluation. As a result, CTOAM imaging data can be collected from preoperative CT imaging tests, sparing patients from further dedicated DXA or qCT scans and accompanying radiation, lowering medical expenditures.

Typically, CT examines the absolute value of bone mineral density in a specific region (ROI) of the body, such as cortical bone and cancellous bone, to assess whether the ROI has reached the indication of osteoporosis. However, in other cases, such as bone grafting, osteotomy and correction, prosthesis implantation, loosening and displacement, a single qCT and DXA diagnosis may not be enough to determine the prognosis developments after surgery. CTOAM, as a reproducible and extremely sensitive CT image post-processing technology, may monitor the distribution of bone tissue with varied densities in the joint surface and assist doctors in choosing the suitable anchor for the prosthesis location. In humans, current studies should focus on the relationship between subchondral bone density and mechanical strength of each articular surface. Emphasis should be given to matching the position of the articular surface’s local high-density area with the position of high mechanical strength to improve the prosthesis’s anchoring stability after implantation. Non-etheless, osteoporotic patients are more likely to experience subsidence, displacement, screw loosening, and iatrogenic fractures during and after spine, shoulder, hip, and knee arthroplasty. It has been demonstrated to some extent that the quality of articular subchondral bone influences the curative impact of surgery. As a result, prior to prosthesis implantation, the density distribution on the articular surfaces should be examined to reduce the risk of revision and secondary surgery. Simultaneously, the device industry should consider models relating to the mechanical strength and bone density distribution of each joint in the human body, in order to provide an assessment reference for relevant prosthesis design and manufacturing engineers as well as clinical orthopaedic surgeons.

In the early days, some scholars combined the subchondral bone mineral density of the articular surface measured by CTOAM technology with the results of articular surface indentation experiments to determine whether there was a correlation between the two. Mühlhofer et al. confirmed the CTOAM and indentation studies early on to see if the two correlate. The study’s findings revealed that the distal tibia’s local maximum density values were not equally distributed and were concentrated in the medial and central areas. The findings of the CTOAM study and the indentation test were similar in that high density values were associated with high mechanical strength; low density values with low mechanical strength. A correlation study found a 0.74–0.97 coefficient of determination link (*R* ^2^) between density data and mechanical strength at each measurement point. The Pearson correlation coefficient was 0.86–0.98 (*p* = 0.05) (Mühlhofer et al., 2009). This study suggested that the ankle endoprosthesis could be anchored not only in the cancellous bone, but also in the subchondral bone, particularly in the distal medial and central regions of the tibia where high-density areas are concentrated, to reduce loosening caused by the prosthesis’s anchoring quality.

Kraljevi et al. discovered that 28 cases of unilateral shoulder glenoid had bicentric high-density distribution, and 4 cases had single-center high-density distribution. The strength test findings revealed that the high mechanical strength area was found in the core of the glenoid’s high-density area (double center and single center). The linear correlation between high-density areas and mechanical strength was 0.62–0.96 (*p* < 0.02), and the coefficient of determination (*R* ^2^) was 0.39–0.91, all of which were statistically significant (Kraljević et al., 2011). Zumstein et al. discovered that the subchondral bone of the articular surface of the humeral head had a high-density distribution in the center (single-center pattern) and anterior-posterior region (double-center pattern), and the distribution of high mechanical strength regions was similar. The median force is 175 N, but the inter-individual variance is substantial, ranging from 10 to 930 N (25%–75% interquartile range, 137–236 N). There is a substantial link between the degree of mineralization and mechanical strength (*p* < 0.01), the correlation coefficient is 0.59–0.96, and the determination coefficient is 0.35–0.93 (Zumstein et al., 2012). Hoechel et al. discovered that the hyperdense area of the acetabular fossa occurred in the anterosuperior region, whereas the hyperdense area of the articular surface of the femoral head appeared in the anterosuperior or posterosuperior region. The findings of the acetabular fossa and femoral head indentation test were comparable to the results of CTOAM postprocessing, however there were still inter-individual variances. High mechanical strength areas were found in the acetabular fossa’s anterosuperior region and the femoral head’s region above the fovea. High mechanical strength was substantially linked with high density regions of the acetabulum and femoral head (SBP correlation coefficients ranged from 0.77 to 0.97 for FL and 0.97 to 0.96 for CF) (Hoechel et al., 2013). Several researchers have discovered a link between the density distribution and mechanical strength of the talar dome (Leumann et al., 2015), cervical vertebral body (Hara et al., 2021), and upper and lower cervical endplate (Orías et al., 2021). Yet, we are aware that, while there is a strong association between the two in this sort of research, a quantitative comparison of bone density and mechanical strength is unachievable. It should be noted that the distribution of subchondral bone density, rather than direct stress measurement, is the direct consequence of CTOAM image measurement. As a result, it may be used as an indirect inference technique for stress changes. The reasoned conclusions, on the other hand, should be referred to and regarded with caution, and realistic biomechanical and clinical examinations are required to validate them.

## 5 Clinical application research

### 5.1 Review on orthopedic surgery

CTOAM technology can evaluate the intra-articular stress change by observing the density changes in bone tissues around the joint. It can be used as a low-cost radiological method imaging research technology for long-term follow-up after surgery (Table 1). For spine and lower limb joint, the stress distribution analyzed by CTOAM technology can reflect the status of degeneration. For no weight-bearing joints like the shoulder, elbow, wrist, the stress distribution can help to estimate the influence of pre- and post-operative, working environment, gender, activity level and movement, ligaments and tendons. Commonly, the changes in bone mineral density only occur 6–12 months after the serious changes in mechanical load.

**TABLE 1:** CTOAM technique evaluate the value of stress abnormal in the different subchondral bone distribution.

Author/year	Name of disease	Positions	CT type	Layer thickness	Number of patients	Age of patients (yrs)	Preoperative evaluate	Postoperative follow-up	References
Lochmuller et al., 1997	unilateral supraspinatus outlet syndrome	Acromion	Picker 1,200	4 mm	Male: 5 Female: 4	52.8 ± 3.3	1. The density maximum is always located at the lateral edge of the acromion, but only 1 case did not detect other maximums	—	Lochmuller et al. (1997)
							2. The dorso-medial acromion has a submaximal, and occasionally the lateral maximum extends to the ventral side of the acromion (types C and D)		
							3. The mineralization types of the affected side and the healthy side were the same in 6 cases, and the mineralization types were different in 3 cases		
							4. The D-type mineralization pattern appears to be related to the hook-like shape of the lower surface of the acromion		
Anetzberger et al., 2002	Tear of the Supraspinatus	Glenoid	Siemens CT	2 mm	Normal: 67 rupture of the supraspinatus muscle: 43	Normal: 71 ± 14 rupture of the supraspinatus muscle: 84 ± 11	1. Among the normal specimens, the anterior and posterior glenoid of the shoulder joint have the highest density	—	Anetzberger et al. (2002)
							2. In some cases, a maximum can be seen in the third area, located in the center area (17%) or antero-inferior area (4%)		
							3. At the site of supraspinatus tendon tear, the third density maximum often occurs in the center of the glenoid (42%)		
Harada et al., 2018	symptomatic rotator cuff tear	Glenoid	Light Speed ultra 16	0.6 mm	32 Male: 19 Female: 13	67.6 ± 8.0	1. The HU in the central area of the glenoid on the affected side was significantly lower than that on the unaffected side	—	Harada et al. (2018)
							2. The HU in the central area of the affected side was significantly lower in patients with larger rotator cuff tears or sub-rotator cuff tendon tears		
Simon et al., 2015	total shoulder arthroplasty subjects with eccentric and concentric wear	Glenoid	GE Lightspeed QZ/i helical scanner	1.25 mm	42	eccentric glenoid wear (65.6 ± 2.4); concentric glenoid wear (66.5 ± 1.6)	1. The SBD of patients with concentric wear type and eccentric wear type was significantly different between different levels	—	Simon et al. (2015)
							2. In the concentric glenoid, the SBD was evenly distributed, with a higher degree of mineralization in the central region, while lower in the posterior, anterior and superior regions		
							3. The distribution of SBD in the eccentric group is uneven, and the mineralization is the largest in the rear part, followed by the lower part		
Deml et al., 2016	chronic posttraumatic shoulder instability; J-shaped bone graft	Glenoid	GE Discovery 750 HD	0.625 mm	14	21–44; mean age, 26.6	On preoperative CT scans, 13 (92.9%) patients had a different mineralization pattern than the contralateral shoulder region distribution	Eleven patients (78.6%) showed a uniform distribution of mineralization on both sides after J-shaped bone grafting. The mineralized type C1 type was similar preoperatively and postoperatively in 1 patient. The mineralization distribution of the 3 patients was different before and after surgery	Deml et al. (2016)
Matsui et al., 2018	osteochondritis dissecans (OCD)	Radial head	Unknown	1 mm	54	13.1 ± 1.7	1. The percentage of anteromedial, posteromedial and anterolateral high-density areas of the radial head fovea was significantly higher than that of the posterolateral area	—	Matsui et al. (2018)
							2. The location and size of the lesion and a history of excessive valgus stress are associated with an imbalance in the fovea of the radial head		
Hasegawa et al., 2020	Ulnar impaction syndrome; ulnar shortening osteotomy	wrist	Unknow	1 mm	Male: 1 Female: 9	Unknown	Severe pain in 5 wrist joints and moderate pain in 5 wrist joints; hyperdense areas of subchondral bone were seen on the distal surface of the ulna	No pain in 3 wrist joints and mild pain in 7 wrist joints; no hyperdense area on the distal surface of the ulna; no significant change in the location of the hyperdense area in the distal radius	Hasegawa et al. (2020)
Hontani et al., 2021	Ulnar impaction syndrome; ulnar shortening osteotomy	distal radioulnar joint	Unknow	1 mm	15	; (Mean age; 47.3)	In L-type, the HDA% in the D area of the sigmoid notch was significantly larger than that in other areas. In type C, the HDA% in the D-V area of the sigmoid notch was significantly larger than that in the dorsal area (D-D and P-D)	The postoperative %HDA in the P-V area of patients with type C was significantly larger than that in the D-D area and tended to be larger than the other two areas (D-V and P-D)	Hontani et al. (2021)
Poilliot et al., 2021	sacroiliac joint dysfunction; Sacroiliac joint arthrodesis	Sacroiliac Joint	conventional CT	0.7–5 mm	18; Male: 9 Female: 9	Mean age; 45 ± 14	The superior zone of the ilium showed significantly higher mineralization values than the corresponding zone of the sacrum	At 3 postoperative follow-ups (≥6 months, ≥12 months, and ≥24 months), a higher degree of mineralization was found to the upper iliac region; Mineral levels all increased in the postoperative state; with regard to the iliac side, there was no significant increase in HU values in any of the subregions at 6, 12, and 24 months	Poilliot et al. (2021)
Iwasaki et al., 2021	medial compartment OA in proximal tibia; high tibial osteotomy	proximal tibia	high-resolution helical CT scanner	1 mm	16	15–30	1. The %HDA of the M2 and M3 regions on the surface of the tibial plateau was significantly higher than that of the control group	1. The medial ratio was significantly reduced to 75.1% (*p* = 0.035 compared to preoperative value); 2. In the medial compartment, HDA increased in the outermost regions of the 4 subregions after HTO, but decreased in the 3 medial subregions	Iwasaki et al. (2021)
							2. The %HDA of all 4 subregions of the lateral ventricle of the OA patients was significantly lower than that of the control group		
Miura et al., 2022	anterior cruciate ligament injury	posteromedial region of the proximal tibia	high-resolution helical CT scanner	1 mm	20	<35	1. The HDA% of the posteromedial region of the ACL deletion group was 7% higher than that of the control group	—	Miura et al. (2022)
							2. The HDA% in the anteromedial region of the ACL deletion group was 6% lower than that of the control group		
							3. In the ACL-deficient group and the control group, there was no significant difference in HDA% between the medial and lateral areas		
Kameda et al., 2021	Medial Open-Wedge High Tibial Osteotomy	Patellofemoral Joint	CT Highspeed Advantage	1 mm	17; Male: 5 Female: 12	Mean age; 54.8	1. The HDA% of the medial notch of the femur is significantly higher than the lateral notch and lateral trochlea of the femur	1. Significantly increased HDA% in the lateral notch and lateral trochlea of the femur and in the medial portion of the lateral facet of the patella; 2. Significant decrease in %HDA in the medial trochlea of the femur and the lateral portion of the lateral facet of the patella	Kameda et al. (2021)
							2. The medial portion of the lateral facet of the patella is significantly higher than the medial facet of the patella		
Matsubara et al., 2022	Knee OA; Medial Opening-Wedge and Lateral Closing-Wedge High Tibial Osteotomy	Distal Tibia	high-resolution helical CT scanner	1 mm	41; Controls:11; OWHTO:18; CWHTO:12	Mean age; Controls: 26; OWHTO: 60.5; CWHTO: 59.1	There was no significant difference in the percentage of HDA in each region of the distal tibia articular surface between the three groups	After OWHTO, area A was significantly increased, and area D was significantly decreased; after CWHTO, area A was significantly decreased, and area C was significantly increased	Matsubara et al. (2022)

#### 5.1.1 Upper limb joint

Rotator cuff injury is a common disease in sports medicine. Clinicians usually pay more attention to the decrease of shoulder function and the soft tissue repair, but concern less to the abnormal stress distribution on the subchondral bone of the cavities glenoidal is. Some authors observed the mineralization of the lower shoulder peak bottom in patients with unilateral subacromial impingement syndrome. They measured the distribution of subchondral bone density at the lower acromion, found that the subacromial bone density of the patients with subacromial impingement syndrome did not increase, which indicated that the increase of subacromial pressure did not induce the increase of subacromial bone mineral density (Lochmuller et al., 1997). It is speculated that the reason for decreasing subacromial mineralization in subacromial impingement syndrome is the decrease of shoulder joint motion due to shoulder joint disease, which is mainly manifested in the obstruction of shoulder joint abduction and the increase of pain level. As a non-weight-bearing joint, the stress distribution change of the shoulder may be connected with the joint instability caused by the shape of the acromion (Neer classification system), surrounding osteophyte, tendon and ligament injury. The rotator cuff muscle group can balance the intra-articular stress of the shoulder. In the condition of rotator cuff injury, especially the supraspinatus injury, the balanced state will break, resulting in poor stability of the shoulder. The compensatory load of the deltoid muscle increased, as the deltoid muscle independently undertaken the shoulder abduction function which was originally joint-maintained by the supraspinatus muscle and deltoid muscle. Some authors have found that the bone mineral density distribution of patients with supraspinatus tears gradually changes from the normal single or dual center mode (anterior upper and posterior upper) to the posterior center and anterior upper center connection mode or three center modes (Anetzberger et al., 2002). The degree and location of supraspinatus muscle injury also determine the balance ability of the supraspinatus muscle and the degree of displacement of the humeral head. This study reported for the first time that the high-density area (HDA) of the glenoid of the shoulder was transferred due to the imbalance of stress distribution in the shoulder joint caused by the tear of the supraspinatus muscle.

The change of glenoid bone mineral density in shoulder arthroplasty (SA) is also a noteworthy research topic. Harada et al. performed CT scanning of bilateral shoulder joints in 32 patients with arthroscopic repair of unilateral rotator cuff tear, and evaluated the distribution of bone mineralization under the shoulder glenoid. They found that the bone mineralization density in the central area of the shoulder glenoid on the affected side was significantly lower than that of the healthy side. In terms of the effect of the size of rotator cuff tear on the degree of bone mineralization, it was found that patients with large area rotator cuff tear had significantly lowered bone mineralization density in the central region of the affected side (Harada et al., 2018). This indicates that the rotator cuff tear of the affected shoulder may reduce the stress load and area distribution in the central area of the glenoid of the shoulder, and the patients who present this kind of distribution usually suffer from a large area rotator cuff tear. In addition, it is also noted that considering that the patients are middle-aged and elderly people. Because the aggregation from the tear and aging of rotator cuff may also lead to an important factor of abnormal stress distribution in the glenoid of the shoulder (Harada et al., 2018).

Total shoulder arthroplasty (TSA) has different wear patterns, which are related to the depth of the replacement joint inserted into the articular surface, the basic bone mass around the prosthesis and the prosthesis type. Some studies used CTOAM technology to observe the distribution of subchondral bone density (SBD) in the glenoid of patients with shoulder osteoarthritis treated with TSA. It was found that there are significant differences in subchondral bone density (SBD) between patients with concentric wear patterns and eccentric wear types. In the concentric shoulder glenoid, SBD distribution is relatively uniform, the mineralization degree in the central area is higher and that in other areas is lower. In the eccentric shoulder glenoid, SBD is unevenly distributed, and the area with highest mineralization degree located in the posterior glenoid, followed by the inferior glenoid (Simon et al., 2015).

Shoulder dislocation is also a common sports injury. Recurrent shoulder dislocation usually involves bone defect of the glenoid. The J-shaped bone transplantation is a classical procedure for treating this case, which fill the defective edge of the glenoid to restore the its normal shape. Deml et al. performed clinical observation and CTOAM measurement on 14 patients with J-shaped bone grafting before and after the operation to evaluate the process of bone reconstruction. In the subsequent postoperative follow-up, they found t 85.7% of patients had an equal distribution of subchondral mineralization in both articular processes (Deml et al., 2016). This study indicated that the postoperative shoulder glenoid adapted to the new intra-articular stress load, resulting in anatomical and mechanical fusion between the bone tissue and bone grafts at the transplantation site, the stress distribution also gradually normalized. According to Wolff’s law, the bone tissue structure can be preserved in the area with sufficient strain. In the low strain region, bone tissue absorbs with time, and the strength and structure of bone tissue decrease. For the shoulder joint, rotator cuff tear not only reduces the stability of the shoulder joint, but also alienates the normal intra-articular stress distribution.

Elbow osteochondritis (OCD) is a common sports injury in adolescent patients. By identifying the abnormal stress distribution of their parts according to imaging classification with the help of CTOAM technology, clinicians can evaluate the pathological status more accurately. Matsui et al. evaluated the elbow of adolescent OCD dissecans with CTOAM images, and explored the relationship between radiologic classification and elbow stress distribution. They found that the percentage of the anterior medial fovea of the radial head, posterior medial and anterior lateral high-density areas were higher than that of posterior lateral areas, the location and size of osteochondritis of the radial head and the history of excessive valgus stress stimulation were correlated with the stress imbalance in the central depression of the radial head (Matsui et al., 2018). Preoperative imaging is helpful for identification of the abnormal stress pattern of the radial head. Especially if the distribution of high-density areas on the outside of the radial head and exfoliative chondritis of the humerus with disorder of the medial epicondyle of the humerus are observed, the risk of late radial humeral arthritis should be considered. Therefore, this study also supported the correlation between the history of excessive valgus stress in the elbow joint and the high-stress distribution in the fovea of the radial head to a certain extent, and illustrated the overloaded stress on the articular surface may reflect the abnormal stress pattern in the radial head joint.

Ulnar impact syndrome (UIS) is defined as painful compression of the ulnar carpal osteofascial compartment due to pathological ulnar variation. This difference may be congenital or acquired, in most cases, it is caused by malunion of distal radius fractures. Due to the shortening of the radius, the load transmitted through the wrist to the ulnar side may increase. Ulnar shortening osteotomy (USO) is a decompression of the operation of the carpal compartment of the ulna, which can reduce the stress distribution on the articular surface of the distal ulna and the distal radius. A study evaluated the distribution of mineralization density of 10 patients who underwent USO due to UIS. Preoperatively, the high-density area (%HDA) of subchondral bone was presented on the distal surface of the ulna in all patients, but no obvious %HDA was found at the last follow-up postoperatively. The %HDA of ulna, radius, navicular fossa and lunate fossa all decreased postoperatively. The analysis of the subchondral bone density distribution showed that USO reduced the stress on the surface of the distal ulna (Hasegawa et al., 2020). The percentage of radius/ulna held an increasing trend, which may due to different mechanisms. For example, the tension on the triangular fibrocartilage complex can absorb the load of the distal ulna surface. Some studies have also evaluated the stress distribution pattern of the distal radioulnar joint (DRUJ) before and after USO for treating UIS. In the linear of the sigmoid notch, the preoperative stress distribution in the DRUJ of the distal radioulnar joint was concentrated on the distal dorsal side, while the postoperative stress distribution is relatively homogeneous. In the arcuate of the sigmoid notch, the preoperative internal stress of the radioulnar joint was concentrated on the distal palmar side, the postoperative stress concentration tended to move to the proximal (Hontani et al., 2021). These results suggest that USO may reduce the risk of degenerative changes in the dorsal side of the sigmoid incision, while the risk of degenerative changes in the proximal palmar side of arcuate the sigmoid incision may increase. Therefore, the morphological evaluation of the sigmoid notch on the transverse plane can predict the location on DRUJ with degenerative changes in patients with accept USO for treating UIS.

#### 5.1.2 Lower limb joints

Sacroiliac joint arthrodesis (SIJ), as the final choice of sacroiliac joint dysfunction (SIJD), can greatly limit the motion of sacroiliac joint and provide lasting pain relief for releasing the tension of sacroiliac joint structure (Buchowski et al., 2005; Foley and Buschbacher, 2006; Murakami et al., 2018a; Poilliot et al., 2021). Surgical SIJ fusion significantly reduced joint motion by more than 50% on the three anatomical planes of the sacroiliac joint (Soriano-Baron et al., 2015). The decrease of sacroiliac joint motion can reduce the pressure of ligaments, muscles and bones around the sacroiliac joint, thereby alleviate SIJ pain to a certain extent (Szadek et al., 2008; Szadek et al., 2010). In addition, SIJ arthrodesis can also change the abnormal loading on auricularis ossis ilii and subchondral bone plate of SIJ, then consequently affecting the mineralization mode. Some authors used CTOAM technology to analyze the bone mineral density of the subchondral bone of SIJ in 18 patients before and after sacroiliac dysfunction. It was found that after surgery, compared with the data of the healthy control cohort (n = 39), the stress and load changes caused by SIJ fixation after arthrodesis led to an increase in the subchondral bone mineral density of the sacrum auricular surface, which was manifested by an increase in the morphological and mechanical integration of the front and lower parts, changes in the overall kinematics of the sacroiliac joint, and different morphological mechanical density patterns (Poilliot et al., 2021). The mineralization changes in sacroiliac joint auricular surface may be related to the surgical approach and screw placement, which provides time-related information about the overall appearance of SIJ and its dysfunction status. As sacroiliac joint fusion changes the stress distribution of the sacroiliac joint and optimizes the mechanical properties of the sacroiliac joint shape, the stress distribution is different from that of normal people, which reflects the adaptability of the human body to the surgery.

Knee osteoarthritis (KOA) and the related malalignment of genu varum change the gait pattern of knee and lower limb kinematics. For example, in the standing phase of gait, the ground reaction force is transmitted from the inner side of the foot to the center of the knee joint, and the medial compartment bears the maximum proportional load (Baliunas et al., 2002; Shelburne et al., 2006). Long-term varus deformity may further aggravate this medial compartment stress distribution. If the deformity is not corrected, the OA will be worsening (Hernigou et al., 1987; Sharma et al., 2001). The common anatomical morphology of KOA with varus deformity includes narrow medial knee joint space of the knee, osteophyte (Teichtahl et al., 2006) and subchondral osteosclerosis. For the single compartment KOA, the medial KOA occupies the most. For end-stage KOA, total knee arthroplasty (TKA) is effective and most recognized treatment. For medial KOA, there are some special surgical options, such as unicompartmental knee arthroplasty (UKA), high tibial osteotomy (HTO) and fibular osteotomy. (Pendleton et al., 2000; van Raaij et al., 2010; McAlindon et al., 2014). The surgery can correct the malalignment thereby reverse the abnormal status of the lower limb, which is of profound biomechanical and clinical significance for treating KOA.

High tibial osteotomy (HTO) surgery (JACKSON and WAUGH, 1961; Liu et al., 2019; Primeau et al., 2021) correct the varus deformity and reduce the load of the medial compartment by laterally shifting the bearing axis. It can release the knee pain, improve the knee function, and delay the progression of KOA. CTOAM technology can observe the pre- and postoperative stress distribution of patients with knee osteoarthritis, and determine the location and severity of KOA through the area of high-density area and upper limit of density of the tibial plateau. Chu et al. found that in the subchondral bone of osteoporosis osteoarthritis (OP-OA), abnormal bone reconstruction leads to the deterioration of bone microstructure and biomechanical properties, potentially affecting the transmission of load stress from cartilage to subchondral bone, accelerating the progression of OA in OP-OA patients (Liu et al., 2019). Therefore, the stress distribution in the knee joint significantly affects the progression of KOA. If the abnormal stress distribution is corrected, the progression of KOA can be delayed to a certain extent. High tibial osteotomy (HTO) surgery (JACKSON and WAUGH, 1961) was developed for correcting varus deformity and reducing the load of the medial compartment by laterally moving the bearing axis. CTOAM technology can observe the preoperative pre- and postoperative stress distribution of patients with KOA in real-time, estimate the location and severity of KOA through the area of high-density area and upper limit of density of the tibial plateau. Iwasaki et al. analyzed the CT data of 16 patients without OA (control group) and 17 patients with KOA before and 1.5 years after HTO. Preoperatively, the medial compartment ratio of %HDA in OA group was significantly higher than that of control group (*p* < 0.001). the medial ratio decreased significantly from preoperative 80.1% to postoperative 75.1% (*p* = 0.035). The change of medial ratio was significantly correlated with the change of hip-knee ankle angle (r = 0.587; *p* = 0.035). In the four subregions of medial compartment, after HTO, %HDA increased in the outermost subregion, and decreased in the three medial subregions (Iwasaki et al., 2021). This indicated HTO can transfer the excessive medial stress to the lateral compartment, thus reducing the stress in the medial compartment of the knee varus knee joint surface, shifting the excessive concentration of the medial compartment to the lateral compartment, and furtherly balancing the medial and lateral stress distribution. Miura et al. analyzed the X-ray and CT data of 20 patients with non-compound injuries of unilateral anterior cruciate ligament (ACL rupture group) and 19 patients with non-ACL injury (control group), %HDA (mean: 21.6%) in the posterior medial area of ACL in the ACL rupture group was significantly higher than that in the control group (14.7%) (*p* = 0.002). In contrast, %HDA (9.4%) in the anterior medial area of ACL in the ACL rupture defect group was significantly lower than that in the control group (15.3%) (*p* = 0.048). The logarithm of the time from ACL injury to CT examination showed a significant correlation with HDA% in the posterior medial region (*p* = 0.032), which indicated that anterior cruciate ligament injury increases the stress in the posterior medial area of the proximal tibia, and long-term condition of ACL deficiency will lead to the accumulation of stress, which will lead to the beginning and progression OA (Miura et al., 2022).

The consistency of patellofemoral joint (PF) and the change of contact stress may lead to patellofemoral osteoarthritis (PFOA) (Goutallier et al., 1979). Some studies have measured the bone mineral density distribution of the femoral trochlear and patellofemoral articular surface of patients who underwent open wedge high tibial osteotomy (OWHTO) before and 1 year after surgery with CTOAM, and found the %HDA percentage of the lateral femoral notch, lateral trochlea and the medial portion of the lateral facet of the patella increased significantly, and the patella height and inclination angle decreased significantly. It shows that the distribution pattern of subchondral bone mineral density of PF articular surface after OWHTO should be laterally shifted compared with the preoperative condition (Kameda et al., 2021). HTO also affect the stress distribution of the ankle. Matsubara et al. used CTOAM technology to analyze the subchondral bone density of distal tibia in patients OWHTO, closed wedge high tibial osteotomy (CWHTO) and unilateral anterior cruciate ligament injury. Preoperatively, no significant difference was found in the %HDA of the distal tibial articular surface between the three groups. Postoperatively, in OWHTO group, the innermost %HDA increased (49.3%–53.0%; *p* = .011), and the outermost %HDA decreased (21.4%–17.2%; *p* = .003). In the CW group, the innermost %HDA of the distal tibial articular surface decreased significantly (55.7%–35.7%; *p* = .001), and the second lateral %HDA increased significantly (23.6%–29.2%; *p* = 0.002) (Matsubara et al., 2022). These results indicated that the distribution pattern of subchondral bone mineral density in the distal tibia shifted medially after OWHTO and laterally after CWHTO. When performing OWHTO on patients with ankle varus, attention should be paid to the inward shifting of ankle stress distribution, which will lead to the deterioration of ankle OA postoperatively.

#### 5.2 Research on sports medicine

Sports training medicine is an emerging subject in recent years, including various fields like surgery, internal medicine and auxiliary diagnosis and treatment. For example, some factors, such as the recurrence of sports injury or the delay in returning to the field, make it difficult for clinicians to find an appropriate balance between the premature return of players and the serious injury of players (Blanch and Gabbett, 2016). According to the data from the London Olympic Games, about 11% of the athletes participating in the Olympic Games will face injuries. During Rio de Janeiro Olympic Games, bone stress injury, muscle, tendon, ligament, and glenoid lip injury were reported. These injuries are mostly seen in the knee joints of lower limbs, ankle joints and shoulder joints of upper limbs, and are most common in track and field athletes. High-intensity sports training and competition are inevitable in Olympic sports. Therefore, it is very important to detect the precursor phenomenon of injury as soon as possible before the stress deformation reaction progresses to stress fracture, and this may be an achievable goal at present (Engebretsen et al., 2013; Murakami et al., 2018b; Hayashi et al., 2018; Heiss et al., 2019). CTOAM technology is also used in guiding sports training and rehabilitation. It can evaluate abnormal bone mineralization caused by long-term stress stimulation in the early stage to predict the force trend of bone and joint according to the stress distribution to prevent injury and correct wrong-training habits (Table 2).

**TABLE 2:** Evaluation of stress distribution in subchondral bone of athletes in different sports by CTOAM technique.

Author/year	Positions	CT type	Layer thickness	Number of patients	Sports project	Age of patients (yrs)	Evaluate results	References
Mochizuki et al., 2005	Glenoid	Lemage Light speed QX/I	2.5 mm	28; Male: 26 Female: 2	Baseball: 25 (pitchers: 18 fielders: 7) handball: 2; volleyball: 1	range, 15–65	1. There were significant differences in mean scores between the two groups in almost all areas except the anterior superior area, the posterior superior area, and the central area	Mochizuki et al. (2005)
							2. In the anterior glenoid area of the shoulder, the average score of the shoulder throwing group was 0.77 points higher than the average score of the control group	
							3. In the anterior inferior glenoid region, the shoulder throwing group was on average 0.68 points higher than the control group	
							4. In the posterior inferior glenoid area of the shoulder joint, the throwing shoulder group was on average 1.06 points higher than the control group	
							5. The DMSB scores of the anterior, anterior inferior, posterior inferior and posterior areas of the shoulder throwing group were significantly higher than those of the control group	
Shimizu et al., 2012	Glenoid	high-resolution helical CT scanner	1 mm	30; male: 30	control group: 10; fielder group:10; pitcher group:10	mean age; control: 22; fielder group: 21; pitcher group: 19	1. A bicentric density distribution pattern across the articular fossa was found in baseball players	Shimizu et al. (2012)
							2. All fielders and pitchers showed greater proportions and a wider distribution of high-density areas in the anterior and posterior glenoid regions compared to controls	
							3. The percentage of high-density area in the total joint socket in the fielder group and the pitcher group was greater than that in the control group	
							4. In the field group, the %HDA of the anterior inferior, posterior inferior and anterior superior glenoid of the shoulder joint was higher than that of the control group	
							5. In the pitchers, the %HDA in the anterior inferior and posterior superior glenoid regions was higher than that in the control group	
							6. The external rotation rate of the shoulder joint of baseball players is greater than that of the control group	
Numaguchi et al., 2021	Glenohumeral Joint	high-resolution helical CT scanner	1 mm	50; male: 50	Baseball, fielders; 11; collegiate pitchers: 12; professional fielders: 15; professional pitchers: 12	mean age, collegiate fielders; 20.3; collegiate pitchers: 21.2; professional fielders: 27.5; professional pitchers: 24.4	1. The stress distribution pattern on the articular surface of the dominant side and the non-dominant side of the collegiate pitchers group was different	Numaguchi et al. (2021)
							2. Also different between the two university groups and the professional pitchers group	
							3. The average HU of the non-dominant side and the dominant side in the collegiate pitchers group was significantly greater than that of the non-dominant side	
							4. On the dominant side, the average HU of the dominant side of both the collegiate pitchers group and the professional pitchers group is greater than the non-dominant side of the professional pitchers group	
Kawasaki et al., 2013	Glenoid	Aquilion CXL	0.5 mm	42; male:42	rugby players:25 (forwards: 10; backs:15) control: 17	mean age, rugby: 21.3; control: 26.2	1. The HU value of the 7th region of the shoulder glenoid of the rugby players is higher than that of the control group	Kawasaki et al. (2013)
							2. The instability of the shoulder joint affected the distribution of HU values in the two groups, and the HU value of the unstable shoulder moved to the lower part of the glenoid of the shoulder	
Momma et al., 2020	Shoulder Joint	Unknow	1 mm	22; male: 22	gymnast group: 12; control: 10	mean age, Gymnast group: 19.4; Control: 20.2	1. There was no significant change in the mean density or distribution pattern of subchondral bone on the dominant and non-dominant sides of gymnasts	Momma et al. (2020)
							2. The stress distribution pattern of the shoulder joint surface of the gymnast and the control group is different	
							3. In gymnasts, areas of high density appear in the posterior-superior region of the articular surface of the humeral head and the anterior-superior and/or posterior-superior regions of the glenoid	
							4. Gymnasts have higher average bone mineral density than control group	
Momma et al., 2019	Wrist	Unknow	Unknow	31; male: 31	gymnast group: 16; control: 15	Unknow	1. In the control group, %HDA was mainly located in lunate fossa and scaphoid fossa	Momma et al. (2019)
							2. In the gymnast group, %HDA is mainly distributed in the dorsal part of the scaphoid fossa, palmar part of the scaphoid fossa and the scaphoid area	
							3 The %HDA values of DSF, PSF and S in the gymnast group were higher than those in the control group	
Momma et al., 2011	Elbow Joint	high-resolution helical CT scanner	1 mm	30, male: 30	Baseball, fielders; 10; pitchers: 10; control: 10	Mean age, Fielders: 20.5; Pitchers: 19; Control: 22.3	1. The area of maximum density for all subjects was located posterior to the distal humeral trochlea	Momma et al. (2011)
							2. The maximum density area of the distal trochlear of the pitcher group was higher than that of the control group	
							3. The pitcher group showed the largest density area distribution in the anterior part of the capitulum	
Momma et al., 2012	Elbow Joint	high-resolution helical CT scanner	1 mm	30, male: 30	Baseball, fielders; 10; pitchers: 10; control: 10	Mean age, Fielders: 21; Pitchers: 19; Control: 22	1. In all subjects, the area of greatest density was located posterior to the trochlea notch	Momma et al. (2012)
							2. Compared with the control group, the %HDA of the trochlea notch in the baseball group was significantly increased	
							3. The largest dense area of the pitcher group is also clearly distributed in the front of the radial head	
Funakoshi et al	Elbow Joint	Unknow	1 mm	30, male: 31	Symptomless Baseball player: 12; UCL insufficiency Baseball player: 12; Control: 7	Symptomless Baseball player: 21.2; UCL insufficiency Baseball player: 19.7; Control: 20.7	1. The high-density areas of the asymptomatic group and the symptomatic group were located in the anterolateral and posteromedial parts of the distal humerus, and the posteromedial parts of the radial head and ulna	Funakoshi et al. (2016)
							2. The high-density areas of the anterior and posteromedial regions of the distal humerus, the radial head and the posteromedial region of the ulna in the baseball group were higher than those in the control group	
							3. In the symptomatic group, the percentage of hyperdense areas in the anterolateral region of the distal humerus and the anterolateral region of the ulna was significantly higher than in the asymptomatic group	
Momma et al	Elbow Joint	Unknow	1 mm	40, male: 40	Baseball, collegiate pitchers: 15; professional pitchers: 13; Control group: 12	Baseball, collegiate pitchers: 20.8; professional pitchers: 23.4; Control group: 22.1	1. The %HDA of the academic and professional groups is located anterior to the capitulum, posterior to the trochlear, and radial head	Momma et al. (2018)
							2. The %HDA of the anterior part of the humeral head, the posterior part of the trochlear, the radial head and the olecranon in the professional group was significantly higher than that in the university group	
Nishida et al	Patellofemoral Joint	high-resolution helical CT scanner	1 mm	40	Baseball, collegiate fielders:10; collegiate pitchers: 10; collegiate soccer:10; control: 10	Baseball, collegiate fielders:21; collegiate pitchers: 19; collegiate soccer: 23; control: 23	The pitcher and fielders groups developed areas of low density proximal to the lateral pulley, while %HDA was seen at the lateral notch, medial notch, and distal end of the medial pulley	Nishida et al. (2012)
Shiota et al	Ankle	Unkown	1 mm	20, male: 20	Control: 10; soccer: 10	Control: 22.9; soccer: 21.6	1. The distribution of HAD can be seen in the distal tibia, proximal talus and distal fibula of football players	Shiota et al. (2020)
							2. The percentage HDA of the ankle joint surface of football players was higher than that of the control group	
Xu et al	Ankle	GE high-resolution helical CT scanner	0.62 mm	20, male:20	Control: 10; taekwondo players: 10	Control: 20.80 ± 0.92; taekwondo players: 21.10 ± 0.88	1. In taekwondo athletes, the percentage of bone tissue volume in the distal tibia and talus with high and medium BMD was significantly higher than that in the control group	Xu et al. (2022)
							2. Taekwondo athletes have high density distribution in the medial area of distal tibia and talus dome	

#### 5.2.1 Upper limb joint

Mochizuki et al. analyzed the bone mineralization of the shoulder glenoid of baseball pitchers, and found that baseball pitchers’ long-term use of throw movement lead to over rotational load from the humeral head on the shoulder glenoid. The displacement of the humeral head due to frequent and rapid abduction external rotation of the humeral head leads to the high-density distribution in the front and rear of the glenoid of the shoulder, laxity and proliferation of the shoulder ligament, and forms a bicentric distribution pattern of the shoulder (Mochizuki et al., 2005). CTOAM can also furtherly diagnose the pathological changes in Bennett (shoulder) of throwing athletes, and help clinical sports medicine experts determine the therapeutic schedule. Shimizu et al. analyzed the bone mineralization of the dominant shoulder joints of 10 baseball fielders, 10 baseball pitchers and 10 non-athletes, and found that the percentage of the high-density area of shoulder joint surface of pitchers and fielders was higher than that of the non-athletes. There was no significant difference in the percentage of shoulder high-density area between baseball pitchers and fielders. Baseball pitchers and fielders have higher shoulder external rotation angles than the non-athletes (Shimizu et al., 2012). The double center high-density area of baseball players’ shoulder joint is due to long-term joint internal stress (compressive stress and shear stress) and excessive joint rotation displacement caused by joint ligament relaxation. Numaguchi et al. analyzed the mineralization of the subchondral bone of the shoulder joint of college baseball players (CP, pitcher group and outfielder) and professional baseball players (PP, pitcher group and outfielder), and found that in the CP Group, the stress distribution pattern on the joint surface of the dominant side and the non-dominant side is different, and the same is true between the two college student groups and the PP group. In the CP Group, the average HU of the humeral head surface on the non-dominant side and the dominant side was greater (*p* = 0.035). On the dominant side, the average HU of humeral head surface and shoulder glenoid in the CP Group was larger than that in the PP group (Numaguchi et al., 2021).

As a high-risk competitive sport, rugby players need to break through the encirclement of their opponents and obtain winning points. Therefore, apart from strong lower limb strength rugby players also need to train strong strength of upper limb and shoulder to break through the encirclement through collision and other forms, which may increase the load of sports shoulder glenoid in long-term training and competition. Kawasaki et al. analyzed the distribution of bone mineral density in the shoulder glenoid of rugby players and found that the area of high bone mineral density in the shoulder glenoid of rugby players is large, and the shoulder joint of rugby players is in a certain unstable state, resulting in a certain degree of degenerative changes in the shoulder joint (such as arthritis and shoulder glenoid lip tear) (Kawasaki et al., 2013).

Gymnastics is a professional competitive sport with high complexity and technical difficulty. Gymnasts usually use high jumping, high somersault, looping and other difficult actions to obtain technical scores, while athletes’ limbs contact with instruments and produce stress during exercise. With the help of the movement inertia and reaction force generated by the action, the enhanced local pressure of athletes’ joints may also be an important factor causing musculoskeletal injuries to athletes. Momma et al. analyzed the bone mineralization of the shoulder joint of male college gymnasts and found that the high-density area appeared in the superior articular surface of the glenohumeral joint (Momma et al., 2020). Because gymnastic athletes bear repeated exercise, high impact load, axial compression, torsion, and tension, accompanied with different degrees of shoulder joint position, athletes are vulnerable to high injury. It is reported that in gymnastic activities, the shoulder can bear a force up to 8.5 times the body weight (Brewin et al., 2000). Momma et al. also analyzed the bone mineralization of the subchondral bone of the wrist joint of college gymnasts. They found that in the control group, the high-density area (%HDAs) was mainly distributed in the lunate fossa (LF) and navicular fossa (SF); In the gymnast group, HDAS is mainly distributed in the dorsal part of the navicular fossa (DSF), the volar part of the navicular fossa (PSF) and the articular surface of the navicular bone (Momma et al., 2019). Repeated gymnastic activities distributed excessive stress through the carpal navicular fossa and the proximal articular surface of the carpal navicular bone.

Elbow injuries occur frequently in throwing sports. To reduce the probability of elbow sports injury, team doctors and coaches should pay attention to any discomfort in the elbow of athletes, especially the worsening pain of baseball pitchers under the corresponding throwing action. Generally, the reason for this kind of high-density differential distribution may be special training, but there are some significant differences in high-density distribution between comprehensive technical training, special technical training, and different training duration.

A study conducted subchondral bone mineralization analysis on the articular surface of the distal humerus of 30 baseball subjects (10 fielders; 10 pitchers; 10 non-athletes). All subjects had high-density areas in the distal trochlea posterior area of the humerus, but the area of high-density areas of baseball players increased more significantly. Besides, high-density distribution containing the part of the capitellum and part of the trochlea was presented in the baseball pitcher group. This subtle differential distribution may due to the surface stress loading between the radial head and the capitulum humeri in the pitching group during the upper arm elevation and acceleration stage of pitching led to the concentration of high-density areas in this part (Momma et al., 2011). However, it is noteworthy that the high-density and concentrated distribution of the capitulum of the distal humerus may be a potential inducing factor of EOCD, which occurs frequently in throwing sports. When baseball pitches, the strength of the elbow will produce a huge valgus and extension overload. This combined overload often leads to acute or chronic injury of the elbow. A specific example is the posterior medial side of the olecranon of the ulna, which is located outside the humeral column at the posterior medial side. Momma et al. observed the distribution of bone mineralization on the articular surface of the ulna olecranon and the radial head of the elbow in baseball players. They found that the high-density area of the entire ulna olecranon and the head of the radius in the baseball group is higher than that in the control group, and the area of the high-density area at the rear of the ulna olecranon and the front of the radial head is more significant (Momma et al., 2012). The findings indicated that baseball pitching activities increase the actual stress on the articular surface at both the rear of the trochlear notch and the front of the radial head. The elbow flexion motion in the throwing action can make the olecranon of the ulna and the trochlear of the humerus come into contact in a large area thereby increasing contact stress. Especially, in the last third of the arm raising stage of the fast-ball and the changing ball throwing, the forearm changes from pronation to supination (Barrentine et al., 1998), which increases the probability of elbow joint injury.

CTOAM technology also explored the stress distribution pattern of symptomatic elbow valgus instability in baseball players. It was found that in the asymptomatic group and the symptomatic group, the high-density areas were in the anterolateral and posteromedial sides of the capitellum of the humerus, as well as the posteromedial and radial heads of the ulna. It indicates that dysfunction with ulnar collateral ligament (UCL) symptoms will produce excessive and accumulated stress in the elbow joint (Funakoshi et al., 2016). If the UCL is insufficient, as the main stabilizer of valgus stress with elbow flexion of 20°–120°, the distal end of ulnar humeral joint will produce high stress in the acceleration stage of throwing action. The functional insufficiency of UCL symptoms will lead to these abnormal stress patterns, which can make baseball pitchers have humeral capitulum exfoliative chondritis or ulnar olecranon stress fracture. Momma et al. found that the high-density area of the distal mobilization elbow joint in the Baseball Academy group and the professional group was mostly found in the anterior part of the glenoid of the scapula, the posterior part of the trochlear, and the head of the radius. The percentage of high-density areas in the anterior part of the capitulum, the posterior part of the trochlear, the radial head and the olecranon in the professional group was significantly higher than that in the college students’ group (Momma et al., 2018).

#### 5.2.2 Lower limb joints

Nishida et al. used CTOAM technology to analyze the bone mineralization of the patellofemoral articular surface of baseball players, and found that low-density areas appeared at the proximal end of the lateral trochlea of fielders, pitchers, and control groups. While at the distal part of the lateral notch both the proximal part of the medial notch and the distal part of the medial femoral trochlea have high-density areas (Nishida et al., 2012), indicating that the proximal part of the lateral trochlea of the femur bears less weight-bearing stress and can be chosen as the best part for autologous osteochondral transplantation for reducing the risk of postoperative symptoms of the patellofemoral joint. This provided an optimized treatment scheme for athletes with osteochondral injury.

Shiota et al. conducted bone mineralization analysis on the ankle joint surface of college football players. It was found that the high-density areas are in the anteromedial and anterolateral parts of the distal tibia, the anteromedial and anterolateral parts of the proximal talus, and the distal fibula (Shiota et al., 2020). The results indicated that the excessive stress in football produces the anterior impact of the ankle joint, which led to the impact of the medial anterolateral front of the ankle joint of football players, forming a high-density distribution. Xu et al. Observed the CTOAM images of the feet of 10 normal people (control group) and 10 high-level Taekwondo athletes. Among Taekwondo athletes, the volume percentage of the distal tibia and talus with high and medium bone density was significantly higher than that of the control group. The force points on the articular surface of the ankle were areas 1, 4, and 7 of the distal tibia and areas 1, 3, 4, and 7 of the talus domes. Taekwondo Athletes’ ankles are comprehensively stressed based on normal stress points to improve the high-density area near the low-stress area (Xu et al., 2022). It is believed that the special stress distribution patterns caused by Taekwondo (such as impact stress, ground reaction force, intra-articular stress), sports technology, lower limb muscle and tendon stress lead to ankle and bone tissue remodeling.

### 6 Advantages and limitation

#### 6.1 Diagnostic accuracy and imaging sensitivity of CTOAM

CTOAM technology is sensitive to water because of its X-ray photons. Due to the high-density, the bone tissue can show an accurate image of stress distribution in joints. Many earlier studies have also confirmed that CTOAM technology has good data consistency with X-rays. Partial volume effect may affect the imaging effect of bone tissue to a certain extent, but it can be effectively resolved through setting the image layer thickness to a thin-layer image (such as 0.5 mm or 0.625 mm), which can improve the image resolution and describe the mineralization in the relatively thin bone plate under the soft bone more accurately (Muller-Gerbl et al., 1990a; Muller-Gerbl et al., 1990b; Eckstein et al., 1995a; Eckstein et al., 1995b). Therefore, CTOAM technology can be used as a reliable method for evaluating of joint bone mineral density and accessing joint internal stress distribution.

#### 6.2 Time and money costs

According to the admission protocol of patients with joint diseases, patients with joint diseases are generally diagnosed by X-ray before admission. If the patient’s X-ray results cannot accurately diagnose joint diseases, CT images, QCT images or MRI will be considered for the next diagnosis, so the time cost of diagnosis will be increased to a certain extent. However, as far as we know, the cost of QCT image and MRI image is high, the amount of ordinary CT image is low, and the results obtained by CTOAM technology are consistent and the image reconstruction efficiency is high. Therefore, CTOAM technology can be added to the daily CT image evaluation as an auxiliary post-treatment method to assist the orthopedic surgeon to estimate the risk of patient’s disease progress, to optimize the treatment plan.

### 6.3 Limitation

Joint stress distribution cannot be directly measured using CTOAM. Only changes in bone density in the subchondral bone are measured by CTOAM. The pressure state of the bone’s articular surface was inferred using Wolff’s law from joint motion and partial material indentation experiments. CTOAM cannot directly measure changes in stress parameters corresponding to high-density regions of the articular surface.

CTOAM technology mainly focuses on the two-dimensional plane area changes in the joint surface of the subjects and specimens, and there is less research on the spatial distribution of intra-articular bone tissue (in the epiphyseal plate), which reduces the spatial visualization of the whole joint.

There are certain differences in the upper and lower limits of bone and joint density CT values (HU) in various parts of the human body. Except for the dark color for high-density pseudo (such as orange, red, dark red or black), the pseudo color distribution (low-density area and medium density area) in other studies is less consistent, and there is a lack of unified color card coding.

As HU is the attenuation coefficient calibrated according to water rather than absolute value, the data difference generated by HU may show different result data according to the difference in CT scanner KV parameters, CT scanner brand, algorithm or inspection conditions, resulting in the lack of repeatability of image measurement data.

The variety of scanning conditions is also a limitation. Due to the differences in bone algorithm and instrument parameters between different instrument manufacturers, there may be a certain degree of data output deviation between different periods, different types of CT scanning instruments, different scanning parameters, and different brands of CT scanning instruments. Of course, if just estimate the overall mode of bone mineral density distribution and the transformation characteristics, this deviation can be reduced to a certain extent by judging the overall trend of bone mineral density distribution and rise and fall changes.

## 7 Conclusion

CTOAM technology is a cost-effective tool technology for studying and diagnosing abnormal stress distribution in the subchondral bone. This method has good sensitivity and specificity in the distribution of subchondral bone stress in the joint. When subchondral bone stress distribution in the articular surface that cannot be identified by conventional X-ray and MRI images, CTOAM technology can be used to make the accurate clinical judgment and prognosis evaluation. This technology can identify the abnormal stress distribution of subchondral bone and osteoarthritis in early athletes and normal people, to achieve the effects of early prevention, early diagnosis, early intervention.
